# Medicine in Spain

**Published:** 1850-08

**Authors:** 


*Medicine in Spain.*—It seems that of late the youth of Spain have manifested so marked a preference for the medical and legal *professions*, and so decided a repugnance at the very name of a *trade*, that the minister of the interior has thought it expedient to circulate the following notice very extensively: "In a social point of view," says the minister, "the country loses, in each of its members thus disgraced, a useful individual; in an economic point of view, society is thus deprived of capital which becomes unfruitful. Meanwhile," adds he, "our sea-ports want pilots, our manufactories want dyers, designers, weavers; we have a lack of men skilled in engineering, in mechanics, &c.; in a word, every branch of industry languishes for want of qualified artisans."—*Union Medicale.*

